# SDF-1α/CXCR4 Signaling in Lipid Rafts Induces Platelet Aggregation via PI3 Kinase-Dependent Akt Phosphorylation

**DOI:** 10.1371/journal.pone.0169609

**Published:** 2017-01-10

**Authors:** Hiroko Ohtsuka, Tomohiro Iguchi, Moyuru Hayashi, Mizuho Kaneda, Kazuko Iida, Motoyuki Shimonaka, Takahiko Hara, Morio Arai, Yuichi Koike, Naomasa Yamamoto, Kohji Kasahara

**Affiliations:** 1 Laboratory of Biomembrane, Tokyo Metropolitan Institute of Medical Science, Tokyo, Japan; 2 Department of Chemistry, Tokyo University of Science, Tokyo, Japan; 3 Stem Cell Project, Tokyo Metropolitan Institute of Medical Science, Tokyo, Japan; 4 Baxalta Japan Ltd., Tokyo, Japan; 5 Department of Drug Metabolism and Clinical Pharmacokinetics, School of Pharmaceutical Sciences, Ohu University, Fukushima, Japan; 6 Department of Biochemistry, School of Pharmaceutical Sciences, Ohu University, Fukushima, Japan; Cornell University, UNITED STATES

## Abstract

Stromal cell-derived factor-1α (SDF-1α)-induced platelet aggregation is mediated through its G protein-coupled receptor CXCR4 and phosphatidylinositol 3 kinase (PI3K). Here, we demonstrate that SDF-1α induces phosphorylation of Akt at Thr308 and Ser473 in human platelets. SDF-1α-induced platelet aggregation and Akt phosphorylation are inhibited by pretreatment with the CXCR4 antagonist AMD3100 or the PI3K inhibitor LY294002. SDF-1α also induces the phosphorylation of PDK1 at Ser241 (an upstream activator of Akt), GSK3β at Ser9 (a downstream substrate of Akt), and myosin light chain at Ser19 (a downstream element of the Akt signaling pathway). SDF-1α-induced platelet aggregation is inhibited by pretreatment with the Akt inhibitor MK-2206 in a dose-dependent manner. Furthermore, SDF-1α-induced platelet aggregation and Akt phosphorylation are inhibited by pretreatment with the raft-disrupting agent methyl-β-cyclodextrin. Sucrose density gradient analysis shows that 35% of CXCR4, 93% of the heterotrimeric G proteins Gαi-1, 91% of Gαi-2, 50% of Gβ and 4.0% of PI3Kβ, and 4.5% of Akt2 are localized in the detergent-resistant membrane raft fraction. These findings suggest that SDF-1α/CXCR4 signaling in lipid rafts induces platelet aggregation via PI3K-dependent Akt phosphorylation.

## Introduction

Lipid rafts are dynamic assemblies of sphingolipids, cholesterol, and proteins that can be stabilized into platforms involved in the regulation of cellular proliferation and differentiation [1]. Lipid rafts have been implicated in signal transduction because various signaling molecules, such as heterotrimeric G proteins, are associated with them [2,3]. A number of studies provide considerable evidence that rafts are integral to the regulation of immune and neuronal signalings. Lipid rafts are also involved in hemostasis and thrombosis. Among blood cells, platelets are critical for maintaining the integrity of the blood coagulation system. Platelet rafts are critical membrane domains in physiological responses such as adhesion and aggregation [4]. The localization of the adhesion receptor GPIb-IX-V complex to membrane rafts is required for platelet adhesion to the vessel wall by binding the von Willebrand factor [5]. Lipid rafts are required for platelet aggregation via the collagen receptor GPVI [6,7], the ADP receptor P2Y12 [8], the Fcγ receptor FcγRIIa [9], and the thromboxane A_2_ receptor [10]. Recently, we have reported that lipid rafts are also required for fibrin clot retraction [11]. Fibrin is translocated to lipid rafts of thrombin-stimulated platelets, and lipid rafts act as platforms where extracellular fibrin and intracellular actomyosin efficiently join via integrin αIIbβ3 to promote outside-in signals, leading to clot retraction.

Platelets are a major source of the chemokine stromal cell-derived factor-1α (SDF-1α, also termed CXCL12), which is stored as part of their α-granule secretome. Platelet-derived SDF-1α modulates paracrine mechanisms such as chemotaxis and differentiation [12]. Platelet-derived SDF-1αenhances recruitment of hematopoietic progenitor cells to the sites of vascular injury and supports their differentiation to endothelial progenitor cells *in vivo* to facilitate vascular remodeling and repair. Platelet-derived SDF-1αis also an autocrine activator of platelets operating through its receptor CXCR4 [13–16]. Pertussis toxin inhibits SDF-1α-induced platelet aggregation, suggesting that its effect is mediated by a pertussis-toxin-sensitive G protein such as Gαi. SDF-1α induces platelet aggregation in a phosphatidylinositol 3 kinase (PI3K)-dependent manner [17]. SDF-1αis highly expressed in atherosclerotic plaques [17], suggesting that SDF-1α-induced platelet aggregation contributes to the pathogenesis of atherosclerosis. Surface expression of SDF-1α on platelets is a prospective biomarker in ischemic events [12]. The SDF-1α expression level on platelets is elevated in patients with acute myocardial infarction [18], acute coronary syndrome [19,20], coronary artery disease [21], valvular aortic stenosis [22], and congestive heart failure [23].

Both SDF-1α and its heterotrimeric G protein-coupled receptor CXCR4 are expressed in the developing cerebellum. SDF-1αalso triggers the chemoattraction of cerebellar granule cells [24]. Previously, we reported that SDF-1α induces transmembrane signaling into cerebellar granule cells via lipid rafts [25]. In this study, we demonstrated that lipid rafts are involved in platelet aggregation and Akt phosphorylation via SDF-1α.

## Materials & Methods

### Materials

The anti-CXCR4 (H118) rabbit polyclonal antibody (sc-9046), anti-Gαi-2 rabbit polyclonal antibody (sc-7276), anti-Gβ rabbit polyclonal antibody (sc-378), and anti-vinculin rabbit polyclonal antibody (sc-5573) were obtained from Santa Cruz Biotechnology (Santa Cruz, CA). The anti-PI3Kβ (C33D4) rabbit monoclonal antibody (#3011), anti-Akt1 (C73H10) rabbit monoclonal antibody (#2938), anti-Akt2 (D6G4) rabbit monoclonal antibody (#3063), and anti-Akt (Pan) (C67E7) rabbit monoclonal antibody (#4691) were purchased from Cell Signaling Technology (Beverly, MA). The anti-Lyn (Lyn8) mouse monoclonal antibody and anti-flotillin-1 mouse monoclonal antibody (F65020) were obtained from Wako (Osaka, Japan) and Transduction Laboratories/BD Pharmingen (Lexington, KY), respectively. The anti-Gαi-1 rabbit polyclonal antibody (371720), and LY294002 were obtained from Merck Millipore Calbiochem (Billerica, MA). Human SDF-1αand AMD3100 were purchased from R&D Systems (Minneapolis, MN). MK-2206 and methyl-β-cyclodextrin (MβCD) were purchased from Selleck Chemicals (Houston, TX) and Sigma-Aldrich (St.Louis, MO), respectively.

### Platelet preparation

Blood was collected into test tubes with 3.8% sodium citrate at a ratio of 9:1. The blood was centrifuged (140 x *g*) to prepare platelet-rich plasma (PRP). To prepare washed platelets, we incubated PRP with 4 mM citric acid and then washed with Tyrode’s buffer (137 mM NaCl, 2.7 mM KCl, 3.75 mM NaH_2_PO_4_, 5 mM N-2-hydroxyethylpiperazine-N’-2-ethanesulfonic acid (HEPES), 0.35% bovine serum albumin, and 5 mM glucose; pH 6.8) containing 1 mM PGE1 and 1 U/ml heparin. Finally, the platelets (3 x 10^8^/ml) were resuspended in Tyrode’s buffer (137 mM NaCl, 2.7 mM KCl, 3.75 mM NaH_2_PO_4_, 5 mM HEPES, 0.35% bovine serum albumin, and 5 mM glucose; pH 7.4) containing 1 mM CaCl_2_ and 1 mM MgCl_2_. The study was approved by the Ethics Committee of Tokyo Metropolitan Institute of Medical Science (No.15–17).

### Measurement of platelet aggregation

Platelet aggregation was measured as the optical density of PRP during aggregation using a platelet aggregometer (NBS HEMA TRACER 601), with the platelet-poor plasma 100% standard for change in optical density.

### Western blot analysis

Freshly isolated human platelets were incubated with SDF-1α and centrifuged for 1 min at 3000 rpm. The resulting pellet was resuspended and boiled in Laemmli sample buffer containing phosphatase inhibitor cocktails 2 and 3 (Sigma-Aldrich). Phosphorylations of myosin light chain 2 (Ser19), Akt (Thr308), Akt (Ser473), PDK1 (Ser241), and GSK3β (Ser9) were detected by western blotting using the phospho-specific mouse monoclonal antibody #3675 and the phospho-specific rabbit monoclonal antibodies #2965, #4058, #3438, and #5558 (Cell Signaling Technology), respectively. Measurement of phosphorylation was performed using ImageJ (National Institutes of Health, Washington, DC). Results are presented as mean +/- standard deviation (SD) of 3 independent experiments.

### Sucrose density gradient analysis

Lipid rafts were obtained as detergent-resistant membranes as previously described [11] with some modifications. Platelets (6 x 10^8^) were homogenized using a Teflon glass homogenizer in 2 ml of TNE/Triton buffer (0.05% Triton X-100, 25 mM Tris-HCl, pH 7.5, 150 mM NaCl, and 1 mM EGTA). The sucrose content of the homogenate was then adjusted to 40% by adding 80% sucrose, placed at the bottom of an ultracentrifuge tube, and overlaid with 35% and then 5% sucrose in 2 ml of TNE without Triton X-100. The discontinuous gradient was centrifuged for 17 h at 39,000 rpm at 4°C in a Hitachi RPS40T rotor (Tokyo, Japan). Lipid rafts accumulating at the 5–35% sucrose interface were carefully collected. The detergent-resistant membrane raft fraction and non-raft fraction (40% sucrose fraction) were analyzed by sodium dodecyl sulfate-polyacrylamide gel electrophoresis (SDS-PAGE) and immunoblotting (n = 2). The percentage of the total in the raft fraction was estimated by densitometry using ImageJ.

### Statistical analyses

Densitometric data were presented as the mean+/- SD of triplicates and analyzed using one-way analysis of variance (ANOVA) followed by a Tukey-Kramer post hoc test in Supporting Information. *P* values <.05 were considered statistically significant.

## Results

### SDF-1α-induced platelet aggregation is mediated by Akt

**Fig 1:**
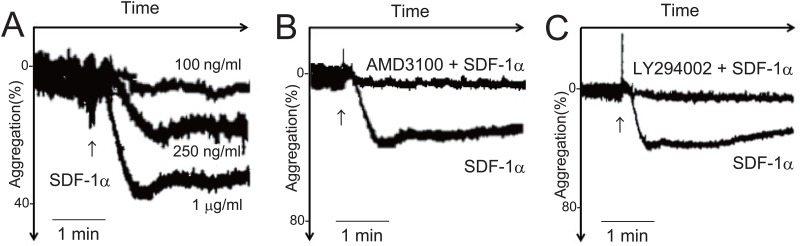
SDF-1α-induced platelet aggregation via CXCR4 and PI3 kinase. (A) Data on platelet aggregation analyzed using platelet-rich plasma. Platelets were stimulated with 100 ng/ml, 250 ng/ml, and 1 μg/ml SDF-1α. (B) 1 μg/ml SDF-1α–induced platelet aggregation with or without pretreatment with 5 μg/ml AMD3100 for 10 min, (C) 1 μg/ml SDF-1α-induced platelet aggregation with or without pretreatment with 30 μM LY294002 for 10 min. Platelet aggregation was measured turbidimetrically using a platelet aggregometer (NBS HEMA TRACER 601) (1 donor, n = 3). Bars represent 1 min. Arrows indicate the time of SDF-1α injection.

SDF-1α induced platelet aggregation in a dose-dependent manner (Fig 1A). The SDF-1α-induced platelet aggregation was inhibited by pretreatment with AMD3100, a CXCR4 antagonist (Fig 1B). Pretreatment with LY294002, a PI3K inhibitor, also inhibited the SDF-1α-induced platelet aggregation (Fig 1C). These observations suggest that SDF-1α-induced platelet aggregation is mediated by the SDF-1α receptor CXCR4 and PI3K, which is consistent with previous findings [17].

**Fig 2:**
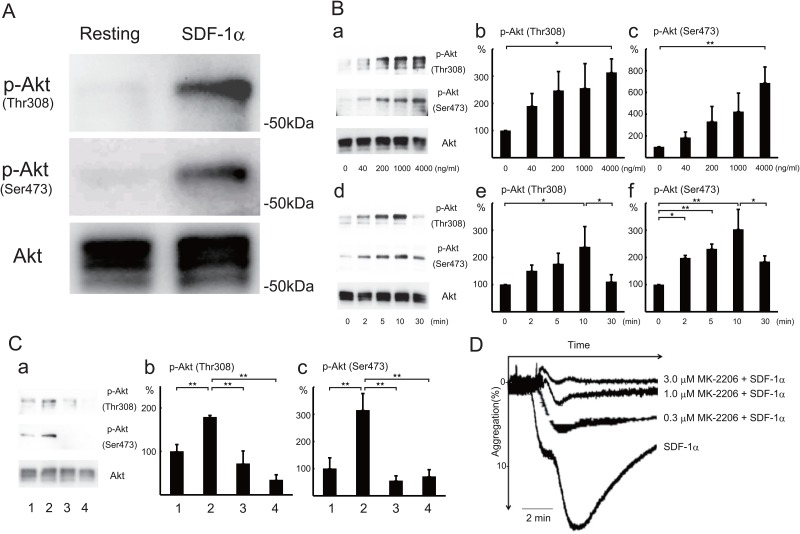
SDF-1α-induced Akt phosphorylation at Thr308 and Ser473 and Akt-dependent platelet aggregation. (A) Washed human platelets were treated with 500 ng/ml SDF-1α for 10 min at 37^°^C. Resting platelets (left lane) and SDF-1α-treated platelets (right lane) were lysed in the Laemmli sample buffer containing phosphatase inhibitors and separated by SDS-PAGE. Phosphorylations of Akt at Thr308 (upper panel), Akt at Ser473 (middle panel), and Akt protein (lower panel) were detected by western blotting using specific Akt antibodies. (B) Dose- and time-dependent phosphorylation of Akt in platelets on SDF-1α treatment. Washed human platelets were treated for 10 min at the indicated concentrations (a-c) or at 500 ng/ml SDF-1α for the indicated times (d-f). Cells were lysed in the Laemmli sample buffer containing phosphatase inhibitors and separated by SDS-PAGE. Phosphorylations of Akt at Thr308 and Ser473 were detected by western blotting (a,d). Phosphorylation of Akt at Thr308 (b,e) and Ser473 (c,f) was quantified by densitometry. Data are presented as the mean +/- SD of triplicates. Statistically significant differences (*P*< .05 shown by *, *P*< .01 shown by **). (C) Effect of CXCR4 antagonist and PI3 kinase inhibitor on SDF-1α-induced Akt phosphorylation. Western blotting (a) and measurement of Akt phosphorylation at Thr308 (b) and Ser473 (c) in resting platelets (lane 1), 500 ng/ml SDF-1α-treated platelets (lane 2), and 500 ng/ml SDF-1α-treated platelets with pretreatment with 5 μg/ml AMD3100 (lane 3), or 30 μM LY294002 (lane 4) for 10 min. Data are presented as the mean +/- SD of triplicates. **Statistically significant differences (*P*< .01). (D) Inhibition of SDF-1α-induced platelet aggregation by Akt inhibitor. SDF-1α(200 ng/ml)-induced platelet aggregation without or with pretreatment with 0.3, 1, or 3 μM MK-2206, an Akt inhibitor, for 10 min (2 donors, n = 3: a single measurement from one donor and a duplicate measurement from another donor on the same day). Bar represents 2 min.

**Fig 3:**
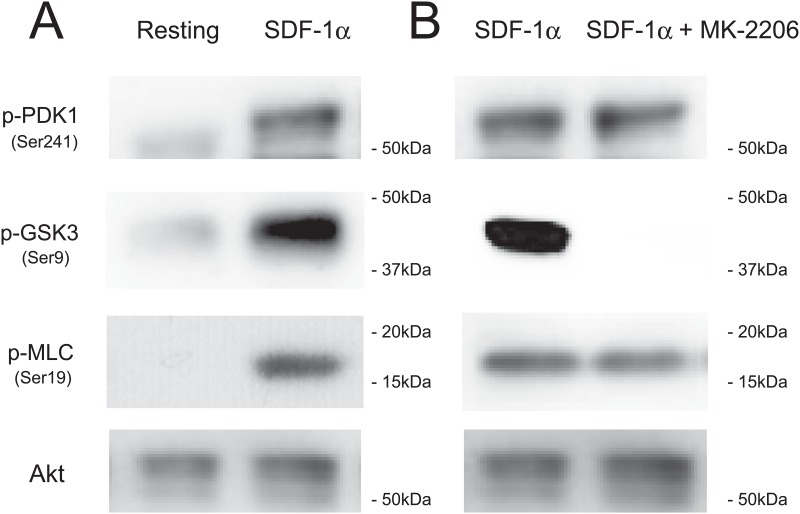
SDF-1α-induced phosphorylation of PDK1, GSK3β, and myosin light chain in platelets. (A) Washed human platelets were treated with 500 ng/ml SDF-1α for 10 min. Resting platelets (left lane) and SDF-1α-treated platelets (right lane) were lysed in the Laemmli sample buffer containing phosphatase inhibitors and separated by SDS-PAGE. Phosphorylations of PDK1 at Ser241 (first panel) and GSK3β at Ser9 (second panel) and myosin light chain (MLC) at Ser19 (third panel) were detected with western blotting. Akt proteins were detected as loading controls (fourth panel). (B) 500 ng/ml SDF-1α-induced phosphorylations of PDK1, GSK3β, and MLC without (left lane) or with pretreatment with 3 μM MK-2206 (right lane).

Serine/threonine kinase Akt (also termed protein kinase B) is activated by G protein-coupled receptors that induce the production of phosphatidylinositol (3,4,5) trisphosphate (PIP_3_) by PI3K [26] These lipids serve as plasma membrane docking sites for pleckstrin-homology domain containing proteins, such as Akt or its upstream activator PDK1. Akt is activated by phosphorylation at Thr308 in the kinase domain by PDK1 [27] and by phosphorylation at Ser473 in the carboxy terminal domain. Akt activation in platelets depends on heterotrimeric G protein Gi signaling pathways [28]. Moreover, a recent report shows that there is also a novel PIP_3_-independent and Gq-dependent Akt translocation mechanism in platelets [29]. Treatment of human platelets with SDF-1α induced the phosphorylation of Akt at Thr308 and Ser473 (Fig 2A). The phosphorylation was dose-dependent (Fig 2Ba, 2Bb and 2Bc) and transient (Fig 2Bd, 2Be and 2Bf) on SDF-1α treatment (Table A in S1 File). The SDF-1α-induced Akt phosphorylation at Thr308 (Fig 2Ca and 2Cb) and Ser473 (Fig 2Ca and 2Cc) were inhibited by pretreatment with AMD3100 (Fig 2C lane 3), or LY294002 (Fig 2C lane 4)(Table B in S1 File). Furthermore, SDF-1α-induced platelet aggregation was inhibited by pretreatment with MK-2206, an Akt inhibitor, in a dose-dependent manner (Fig 2D). These observations suggest that SDF-1 α-induced platelet aggregation is mediated via Akt activation. Consistent with this idea, SDF-1α induced phosphorylation of PDK1 on the activation loop Ser241 and GSK3β at Ser9 (a downstream substrate of Akt [27]) and myosin light chain (MLC) at Ser19 (a downstream element of the PI3K/Akt signaling pathway in platelets [30]) (Fig 3A). The SDF-1α-induced phosphorylation of GSK3β, but not PDK1 and MLC, was inhibited by pretreatment with MK-2206 (Fig 3B). The MLC phosphorylation might be due to multiple signaling pathways.

### Lipid rafts are involved in SDF-1α-induced platelet aggregation and Akt phosphorylation

**Fig 4:**
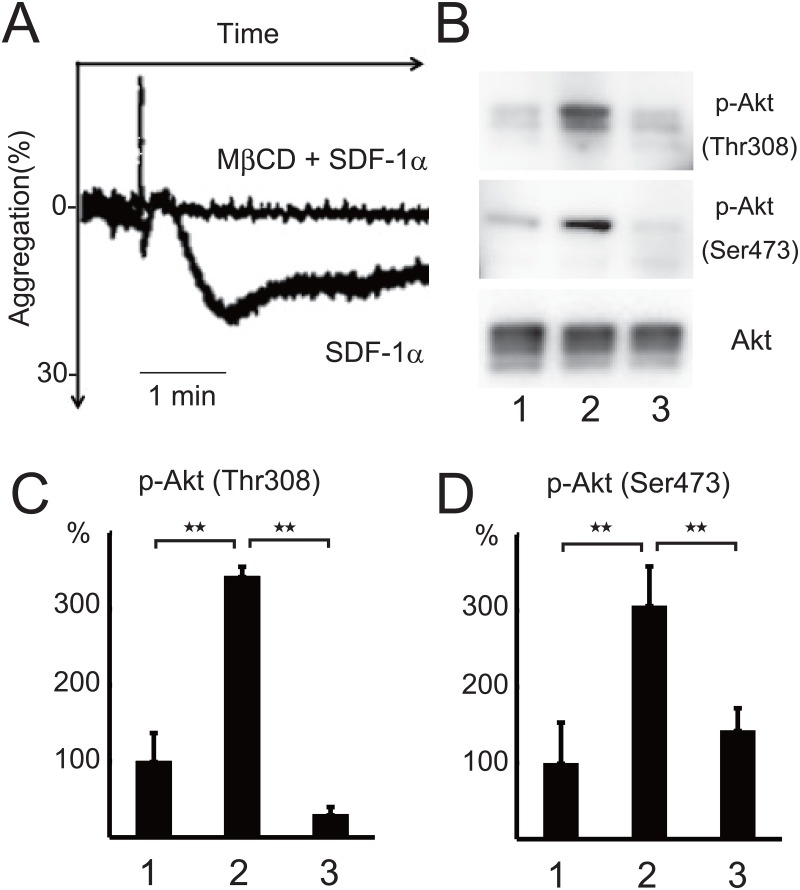
Inhibition of SDF-1α-induced platelet aggregation and Akt phosphorylation by raft disruption with methyl-β-cyclodextrin. (A) 500 ng/ml SDF-1α-induced platelet aggregation with or without pretreatment with 2% methyl-β-cyclodextrin (MβCD) for 15 min (2 donors, n = 4: a duplicated measurement from 2 donors on the same day). Bar represents 1 min. Western blotting (B) and measurement of Akt phosphorylation at Thr308 (C) and Ser473 (D) in resting platelets (lane 1), 500 ng/ml SDF-1α-treated platelets (lane 2), and 500 ng/ml SDF-1α-treated platelets with pretreatment with 2% MβCD for 15 min (lane 3). Phosphorylation was quantified by densitometry. Data are presented as the mean +/- SD of triplicates. **Statistically significant difference (*P*< .01).

**Fig 5:**
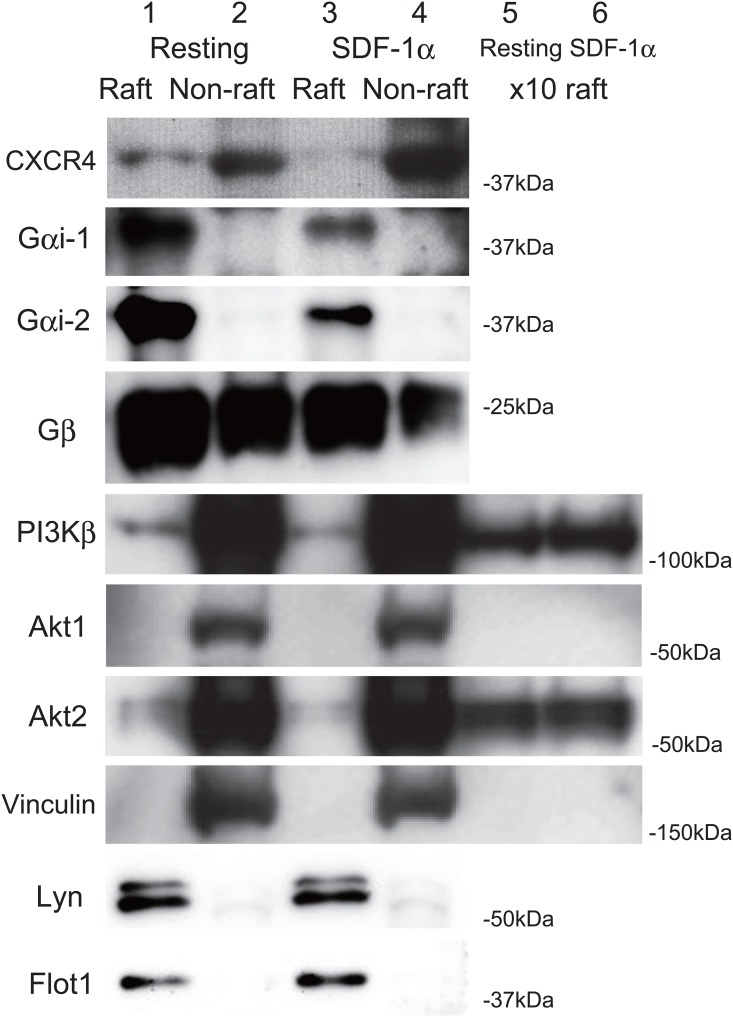
Distribution of SDF-1α-mediated signaling molecules in raft and non-raft fractions. The raft (lanes 1,3) and non-raft (lanes 2,4) fractions and 10-fold increased amount of raft fractions (lanes 5,6) of resting (lanes 1,2,5) and SDF-1α-treated (lanes 3,4,6) platelets were subjected to SDS-PAGE. CXCR4, Gαi-1, Gαi-2, Gβ, PI3Kβ, Akt1, Akt2, and vinculin were detected by western blotting using specific antibodies. Lyn and flotillin-1 (Flot1) are marker proteins of lipid rafts [53,31].

Lipid rafts are involved in signal transduction by several G protein-coupled receptors such as the thrombin receptor PAR1, the ADP receptor P2Y12 and the thromboxane A_2_ receptor in platelets [31,8,10]. The pretreatment of platelets with the cholesterol-depleting and raft-disrupting agent MβCD [11] inhibited the SDF-1α-induced platelet aggregation (Fig 4A) and Akt phosphorylation at Thr308 (Fig 4B and 4C), Ser473 (Fig 4B and 4D)(Table C in S1 File). These observations suggest that lipid rafts are mediated in SDF-1α-induced platelet aggregation and Akt activation. To investigate whether SDF-1α-mediated signaling molecules exist in lipid rafts, we isolated the lipid raft fraction by treating platelets with cold Triton X-100 and separating the detergent-resistant membrane by sucrose density gradient centrifugation. Fig 5 shows the distribution of CXCR4 and heterotrimeric G proteins on the raft (lanes 1,3,) and non-raft fractions (lanes 2,4) of resting (lanes 1,2) and SDF-1α-treated platelets (lanes 3,4). The signals for PI3Kβ and Akt2 in the raft fractions were weakly identified. To determine clearly the presence of those proteins in the raft fraction, 10-fold increased amount of the resting and SDF-1α-treated raft fractions were also immunoblotted.　The clear bands of PI3Kβ and Akt2, but not Akt1 and vinculin, were detected in the raft fractions of resting (lane 5) and SDF-1α-treated platelets (lane 6). The summary of the data obtained by densitometry: 35% and 13% of CXCR4, 93% and 93% of Gαi-1, 91% and 89% of Gαi-2, 50% and 62% of Gβ, 4.0% and 4.1% of PI3Kβ, 4.5% and 4.1% of Akt2 are localized in the raft fraction of resting and SDF-1α-treated platelets, respectively. Taken together, these results suggest that CXCR4-signaling by SDF-1α on lipid rafts leading to platelet aggregation occurs through PI3K-dependent Akt phosphorylation.

## Discussion

In the present study, we demonstrated that the chemokine SDF-1α induces platelet aggregation and Akt phosphorylation at Thr308 and Ser473 through CXCR4 and PI3K. The SDF-1α-induced platelet aggregation is inhibited by the Akt inhibitor MK-2206, suggesting that SDF-1 α-induced platelet aggregation is mediated by Akt activation. There is substantial evidence suggesting that Akt has an indirect role in regulating platelet aggregation [32]. Our observations suggest that SDF-1α induces Akt activation and phosphorylation of GSK3β, a putative Akt effector. Akt-mediated phosphorylation of GSK3β at Ser9 inhibits it’s constitutive kinase activity. Mouse platelets lacking a single allele of GSK3β are hypersensitive to agonist-induced aggregation and secretion, suggesting that GSK3β plays a negative role in regulating platelet activation [33]. Therefore, SDF-1α-induced GSK3β phosphorylation at Ser9 might contribute to platelet aggregation. However, precise mechanism of SDF-1α-induced platelet aggregation via Akt remains to be explored.

Lipid rafts are the major determinant for correct chemokine receptor function [34]. Incorporation of CXCR4 into lipid rafts is responsible for the homing effect in human bone marrow CD34+ cells [35]. SDF-1αinduces CXCR4 association with flotillin-1 in lipid rafts of T cells [36]. Flotillin-1, a raft-associated integral membrane protein, a putative adapter protein recruiting signaling molecules. Down-regulation of flotillin-1 expression using RNAi technology inhibits SDF-1α/CXCR4 signaling and function. The PI3K/Akt pathway is compartmentalized within lipid rafts [37]. Production of PIP_3_ in platelet rafts is critical in platelet activation [38]. Lipid rafts play a crucial role in triggering the Akt signaling pathway by facilitating Akt recruitment and activation in the plasma membrane [39]. Lipid raft disruption impairs the Akt signaling pathway in epidermal keratinocytes [40]. Suppressing the formation of raft-associated Akt inhibits SDF-1α-induced invasion of esophageal carcinoma cells [41]. In the present study, we demonstrated that SDF-1α-induced platelet aggregation and Akt phosphorylation are inhibited by the raft disrupting agent MβCD. Sucrose density gradient analysis showed that CXCR4, heterotrimeric G proteins, and part of PI3K, Akt are present in platelet detergent-resistant membrane rafts, suggesting that lipid rafts are involved in Akt-mediated platelet aggregation by SDF-1α.

Three mammalian Akt isoforms, Akt1, Akt2, and Akt3, are expressed in platelets [32,42]. Akt1(-/-) platelets exhibit impaired platelet responses to thrombin and collagen. Akt2(-/-) platelets exhibit impaired platelet responses to thrombin receptor activated peptide. Akt3(-/-) platelets exhibit impaired platelet responses to low concentrations of thrombin receptor activated peptide and thromboxane A_2_. SDF-1α/CXCR4 signaling activates Akt1 in the lipid rafts of prostate cancer cells [43]. PI3K/Akt2 signaling in lipid rafts is involved in enterocyte differentiation [44]. Raft-mediated PKC zeta/Akt3 signaling is involved in vascular smooth muscle cell growth arrest [45]. Our study demonstrated significant amounts of PI3Kβ and Akt2 in the raft fraction of platelets, suggesting that raft-mediated PI3Kβ/Akt2 signaling may be involved in SDF-1α-induced platelet aggregation.

We have investigated the functions of sphingolipids in raft-mediated signal transduction using platelets and neurons [2,3,11,25,46–52]. SDF-1αinduces the activation and translocation of the heterotrimeric G protein Goα to ganglioside GD3-rich rafts, resulting in growth cone collapse of cerebellar granule neurons [25]. The growth cone collapse is prevented by the raft-disrupting agent MβCD. We also demonstrated that sphingomyelin in platelet rafts is involved in outside-in signals, leading to clot retraction [11]. Thrombin induces the translocation of fibrin to sphingomyelin-rich rafts of platelets and enhances outside-in signals, contributing to efficient clot retraction. Furthermore, clot retraction of sphingomyelin-rich raft-depleted platelets from sphingomyelin synthase knockout mice is delayed. The nature of sphingolipids involved in the SDF-1α/CXCR4 pathway in platelets is the subject of future research.

## Supporting Information

S1 File Statistical analysis of dose- and time-dependent phosphorylation of Akt in platelets on SDF-1α treatment (Table A). Statistical analysis of effect of CXCR4 antagonist and PI3 kinase inhibitor on SDF-1α-induced Akt phosphorylation (Table B). Statistical analysis of inhibition of SDF-1α-induced Akt phosphorylation by raft disruption with methyl-β-cyclodextrin (Table C).
(PDF)

## Abbreviations

MLC: myosin light chain
MβCD: methyl-β-cyclodextrin
PI3K: phosphatidylinositol 3 kinase
PIP_3_: phosphatidylinositol (3,4,5) trisphosphate
PRP: platelet-rich plasma
SDF-1α: stromal cell-derived factor-1α
SDS-PAGE: sodium dodecyl sulfate-polyacrylamide gel electrophoresis
RNAi: RNA interference
WASP: Wiskott-Aldrich syndrome protein

## References

[1] Simons K, Gerl MJ. Revitalizing membrane rafts: new tools and insights. Nat Rev Mol Cell Biol 2010; 11(10): 688–99. 10.1038/nrm2977 20861879

[2] Kasahara K, Sanai Y. Functional roles of glycosphingolipids in signal transduction via lipid rafts. Glycoconj J 2000; 17(3–4): 153–62. 11201786 10.1023/a:1026576804247

[3] Kasahara K, Sanai Y. Possible roles of glycosphingolipids in lipid rafts. Biophys Chem 1999; 82(2–3): 121–7. 10631795 10.1016/s0301-4622(99)00111-8

[4] Bodin S, Tronchère H, Payrastre B. Lipid rafts are critical membrane domains in blood platelet activation processes. Biochim Biophys Acta 2003; 1610(2): 247–57. 12648778 10.1016/s0005-2736(03)00022-1

[5] Shrimpton CN, Borthakur G, Larrucea S, Cruz MA, Dong JF, López JA. Localization of the adhesion receptor glycoprotein Ib-IX-V complex to lipid rafts is required for platelet adhesion and activation. J Exp Med 2002; 196(8): 1057–66. 10.1084/jem.20020143 12391017 PMC2194038

[6] Locke D, Chen H, Liu Y, Liu C, Kahn ML. Lipid rafts orchestrate signaling by the platelet receptor glycoprotein VI. J Biol Chem 2002; 277(21): 18801–9. 10.1074/jbc.M111520200 11844795

[7] Ezumi Y, Kodama K, Uchiyama T, Takayama H. Constitutive and functional association of the platelet collagen receptor glycoprotein VI-Fc receptor gamma-chain complex with membrane rafts. Blood 2002; 99(9): 3250–5. 11964290 10.1182/blood.v99.9.3250

[8] Quinton TM, Kim S, Jin J, Kunapuli SP. Lipid rafts are required in Galpha(i) signaling downstream of the P2Y12 receptor during ADP-mediated platelet activation. J Thromb Haemost 2005; 3(5): 1036–41. 10.1111/j.1538-7836.2005.01325.x 15869601

[9] Bodin S, Viala C, Ragab A, Payrastre B. A critical role of lipid rafts in the organization of a key FcgammaRIIa-mediated signaling pathway in human platelets. Thromb Haemost 2003; 89(2): 318–30. 12574813

[10] Moscardó A, Vallés J, Latorre A, Santos MT. The association of thromboxane A2 receptor with lipid rafts is a determinant for platelet functional responses. FEBS Lett 2014; 588(17): 3154–9. 10.1016/j.febslet.2014.06.057 24996187

[11] Kasahara K, Kaneda M, Miki T, Iida K, Sekino-Suzuki N, Kawashima I, et al Clot retraction is mediated by factor XIII-dependent fibrin-αIIbβ3-myosin axis in platelet sphingomyelin-rich membrane rafts. Blood 2013; 122(19): 3340–8. 10.1182/blood-2013-04-491290 24002447

[12] Chatterjee M, Gawaz M. Platelet-derived CXCL12 (SDF-1α): basic mechanisms and clinical implications. J Thromb Haemost 2013; 11(11): 1954–67. 10.1111/jth.12404 24024928

[13] Kowalska MA, Ratajczak J, Hoxie J, Brass LF, Gewirtz A, Poncz M, et al Megakaryocyte precursors, megakaryocytes and platelets express the HIV co-receptor CXCR4 on their surface: determination of response to stromal-derived factor-1 by megakaryocytes and platelets. Br J Haematol 1999; 104(2): 220–9. 10050701 10.1046/j.1365-2141.1999.01169.x

[14] Kowalska MA, Ratajczak MZ, Majka M, Jin J, Kunapuli S, Brass L, et al Stromal cell-derived factor-1 and macrophage-derived chemokine: 2 chemokines that activate platelets. Blood 2000; 96(1): 50–7. 10891429

[15] Chatterjee M, Seizer P, Borst O, Schönberger T, Mack A, Geisler T, et al SDF-1α induces differential trafficking of CXCR4-CXCR7 involving cyclophilin A, CXCR7 ubiquitination and promotes platelet survival. FASEB J 2014; 28(7): 2864–78. 10.1096/fj.14-249730 24668750

[16] Walsh TG, Harper MT, Poole AW. SDF-1α is a novel autocrine activator of platelets operating through its receptor CXCR4. Cell Signal 2015; 27(1): 37–46. 10.1016/j.cellsig.2014.09.021 25283599 PMC4265729

[17] Abi-Younes S, Sauty A, Mach F, Sukhova GK, Libby P, Luster AD. The stromal cell-derived factor-1 chemokine is a potent platelet agonist highly expressed in atherosclerotic plaques. Circ Res 2000; 86(2): 131–8. 10666407 10.1161/01.res.86.2.131

[18] Geisler T, Fekecs L, Wurster T, Chiribiri A, Schuster A, Nagel E, et al Association of platelet-SDF-1 with hemodynamic function and infarct size using cardiac MR in patients with AMI. Eur J Radiol 2012; 81(4): e486–90. 10.1016/j.ejrad.2011.06.019 21724347

[19] Stellos K, Bigalke B, Langer H, Geisler T, Schad A, Kögel A, et al Expression of stromal-cell-derived factor-1 on circulating platelets is increased in patients with acute coronary syndrome and correlates with the number of CD34+ progenitor cells. Eur Heart J 2009; 30(5): 584–93. 10.1093/eurheartj/ehn566 19109356

[20] Wurster T, Stellos K, Haap M, Seizer P, Geisler T, Otton J, et al Platelet expression of stromal-cell-derived factor-1 (SDF-1): an indicator for ACS? Int J Cardiol 2013; 164(1): 111–5. 10.1016/j.ijcard.2011.06.082 21737155

[21] Stellos K, Rahmann A, Kilias A, Ruf M, Sopova K, Stamatelopoulos K, et al Expression of platelet-bound stromal cell-derived factor-1 in patients with non-valvular atrial fibrillation and ischemic heart disease. J Thromb Haemost 2012; 10(1): 49–55. 10.1111/j.1538-7836.2011.04547.x 22044645

[22] Wurster T, Tegtmeyer R, Borst O, Rath D, Geisler T, Gawaz M, et al Platelet expression of stromal cell-derived factor-1 is associated with the degree of valvular aortic stenosis. PLoS One 2014; 9(5): e97405 10.1371/journal.pone.0097405 24834915 PMC4023969

[23] Jorbenadze R, Schleicher E, Bigalke B, Stellos K, Gawaz M. Expression of platelet-bound stromal-cell derived factor-1 (SDF-1) and number of CD34(+) progenitor cells in patients with congestive heart failure. Platelets 2014; 25(6): 409–15. 10.3109/09537104.2013.829913 24102302

[24] Ma Q, Jones D, Borghesani PR, Segal RA, Nagasawa T, Kishimoto T, et al Impaired B-lymphopoiesis, myelopoiesis, and derailed cerebellar neuron migration in CXCR4- and SDF-1-deficient mice. Proc Natl Acad Sci U S A 1998; 95(16): 9448–53. 9689100 10.1073/pnas.95.16.9448 PMC21358

[25] Yuyama K, Sekino-Suzuki N, Sanai Y, Kasahara K. Translocation of activated heterotrimeric G protein Galpha(o) to ganglioside-enriched detergent-resistant membrane rafts in developing cerebellum. J Biol Chem 2007; 282(36): 26392–400. 10.1074/jbc.M705046200 17623667

[26] Fayard E, Xue G, Parcellier A, Bozulic L, Hemmings BA. Protein kinase B (PKB/Akt), a key mediator of the PI3K signaling pathway. Curr Top Microbiol Immunol 2010; 346: 31–56. 10.1007/82_2010_58 20517722

[27] Dangelmaier C, Manne BK, Liverani E, Jin J, Bray P, Kunapuli SP. PDK1 selectively phosphorylates Thr(308) on Akt and contributes to human platelet functional responses. Thromb Haemost 2014; 111(3): 508–17. 10.1160/TH13-06-0484 24352480 PMC4079046

[28] Kim S, Jin J, Kunapuli SP. Akt activation in platelets depends on Gi signaling pathways. J Biol Chem 2004; 279(6): 4186–95. 10.1074/jbc.M306162200 14623889

[29] Badolia R, Manne BK, Dangelmaier C, Chernoff J, Kunapuli SP. Gq-dependent Akt translocation to the membrane: a novel PIP_3_-independent mechanism in platelets. Blood 2015; 125(1): 175–84. 10.1182/blood-2014-05-576306 25331114 PMC4281826

[30] Signorello MG, Leoncini G. Effect of 2-arachidonoylglycerol on myosin light chain phosphorylation and platelet activation: The role of phosphatidylinositol 3 kinase/AKT pathway. Biochimie 2014; 105: 182–91. 10.1016/j.biochi.2014.07.014 25068972

[31] Zhang Y, Wang Y, Xiang Y, Lee W. Prohibitins are involved in protease-activated receptor 1-mediated platelet aggregation. J Thromb Haemost 2012; 10(3): 411–8. 10.1111/j.1538-7836.2011.04607.x 22212092

[32] Woulfe DS. Akt signaling in platelets and thrombosis. Expert Rev Hematol 2010; 3(1): 81–91. 10.1586/ehm.09.75 20352060 PMC2844717

[33] Li D, August S, Woulfe DS. GSK3β is a negative regulator of platelet function and thrombosis. Blood 2008; 111(7):3522–3530. 10.1182/blood-2007-09-111518 18218855 PMC2275019

[34] Mañes S, Lacalle RA, Gómez-Moutón C, del Real G, Mira E, Martínez-A C. Membrane raft microdomains in chemokine receptor function. Semin Immunol 2001; 13(2): 147–57. 10.1006/smim.2000.0306 11308298

[35] Wysoczynski M, Reca R, Ratajczak J, Kucia M, Shirvaikar N, Honczarenko M, et al Incorporation of CXCR4 into membrane lipid rafts primes homing-related responses of hematopoietic stem/progenitor cells to an SDF-1 gradient. Blood 2005; 105(1): 40–8. 10.1182/blood-2004-04-1430 15328152

[36] Giri B, Dixit VD, Ghosh MC, Collins GD, Khan IU, Madara K, et al CXCL12-induced partitioning of flotillin-1 with lipid rafts plays a role in CXCR4 function. Eur J Immunol 2007; 37(8): 2104–16. 10.1002/eji.200636680 17634952 PMC2271046

[37] Gao X, Lowry PR, Zhou X, et al PI3K/Akt signaling requires spatial compartmentalization in plasma membrane microdomains. Proc Natl Acad Sci U S A 2011; 108(35): 14509–14. 10.1073/pnas.1019386108 21873248 PMC3167518

[38] Bodin S, Giuriato S, Ragab J, Humbel BM, Viala C, Vieu C, et al Production of phosphatidylinositol 3,4,5-trisphosphate and phosphatidic acid in platelet rafts: evidence for a critical role of cholesterol-enriched domains in human platelet activation. Biochemistry 2001; 40(50): 15290–9. 11735411 10.1021/bi0109313

[39] Lasserre R, Guo XJ, Conchonaud F, Hamon Y, Hawchar O, Bernard AM, et al Raft nanodomains contribute to Akt/PKB plasma membrane recruitment and activation. Nat Chem Biol 2008; 4(9): 538–47. 10.1038/nchembio.103 18641634

[40] Calay D, Vind-Kezunovic D, Frankart A, Lambert S, Poumay Y, Gniadecki R. Inhibition of Akt signaling by exclusion from lipid rafts in normal and transformed epidermal keratinocytes. J Invest Dermatol 2010; 130(4): 1136–45. 10.1038/jid.2009.415 20054340

[41] Lin ML, Lu YC, Chen HY, Lee CC, Chung JG, Chen SS. Suppressing the formation of lipid raft-associated Rac1/PI3K/Akt signaling complexes by curcumin inhibits SDF-1α-induced invasion of human esophageal carcinoma cells. Mol Carcinog 2014; 53(5): 360–79. 10.1002/mc.21984 23192861

[42] O'Brien KA, Stojanovic-Terpo A, Hay N, Du X. An important role for Akt3 in platelet activation and thrombosis. Blood 2011; 118(15): 4215–23. 10.1182/blood-2010-12-323204 21821713 PMC3204738

[43] Chinni SR, Sivalogan S, Dong Z, Filho JC, Deng X, Bonfil RD, et al CXCL12/CXCR4 signaling activates Akt-1 and MMP-9 expression in prostate cancer cells: the role of bone microenvironment-associated CXCL12. Prostate 2006; 66(1): 32–48. 10.1002/pros.20318 16114056

[44] Li X, Leu S, Cheong A, Zhang H, Baibakov B, Shih C, et al Akt2, phosphatidylinositol 3-kinase, and PTEN are in lipid rafts of intestinal cells: role in absorption and differentiation. Gastroenterology 2004; 126(1): 122–35. 14699494 10.1053/j.gastro.2003.10.061

[45] Fox TE, Houck KL, O'Neill SM, Nagarajan M, Stover TC, Pomianowski PT, et al Ceramide recruits and activates protein kinase C zeta (PKC zeta) within structured membrane microdomains. J Biol Chem 2007; 282(17): 12450–7. 10.1074/jbc.M700082200 17308302

[46] Kasahara K, Watanabe Y, Yamamoto T, Sanai Y. Association of Src family tyrosine kinase Lyn with ganglioside GD3 in rat brain. Possible regulation of Lyn by glycosphingolipid in caveolae-like domains. J Biol Chem 1997; 272(47): 29947–53. 9368072 10.1074/jbc.272.47.29947

[47] Kasahara K, Watanabe K, Takeuchi K, Kaneko H, Oohira A, Yamamoto T, et al Involvement of gangliosides in glycosylphosphatidylinositol-anchored neuronal cell adhesion molecule TAG-1 signaling in lipid rafts. J Biol Chem 2000; 275(44): 34701–9. 10.1074/jbc.M003163200 10944523

[48] Kasahara K, Watanabe K, Kozutsumi Y, Oohira A, Yamamoto T, Sanai Y. Association of GPI-anchored protein TAG-1 with src-family kinase Lyn in lipid rafts of cerebellar granule cells. Neurochem Res 2002; 27(7–8): 823–9. 12374219 10.1023/a:1020265225916

[49] Yuyama K, Sekino-Suzuki N, Yamamoto N, Kasahara K. Ganglioside GD3 monoclonal antibody-induced paxillin tyrosine phosphorylation and filamentous actin assembly in cerebellar growth cones. J Neurochem 2011; 116(5): 845–50. 10.1111/j.1471-4159.2010.07071.x 21214573

[50] Sekino-Suzuki N, Yuyama K, Miki T, Kaneda M, Suzuki H, Yamamoto N, et al Involvement of gangliosides in the process of Cbp/PAG phosphorylation by Lyn in developing cerebellar growth cones. J Neurochem 2013; 124(4): 514–22. 10.1111/jnc.12040 23035659

[51] Miki T, Kaneda M, Iida K, Hasegawa G, Murakami M, Yamamoto N, et al An anti-sulfatide antibody O4 immunoprecipitates sulfatide rafts including Fyn, Lyn and the G protein α subunit in rat primary immature oligodendrocytes. Glycoconj J 2013; 30(9): 819–23. 10.1007/s10719-013-9487-5 23877649

[52] Kasahara K, Souri M, Kaneda M, Miki T, Yamamoto N, Ichinose A. Impaired clot retraction in factor XIII A subunit-deficient mice. Blood 2010; 115(6): 1277–9. 10.1182/blood-2009-06-227645 19996413

[53] Dorahy DJ, Burns GF. Active Lyn protein tyrosine kinase is selectively enriched within membrane microdomains of resting platelets. Biochem J 1998; 333(Pt2): 373–9. 9657978 10.1042/bj3330373 PMC1219595

