# Supplementary material for: SDF-1α/CXCR4 Signaling in Lipid Rafts Induces Platelet Aggregation via PI3 Kinase-Dependent Akt Phosphorylation
Source: PLoS One. 2017 Jan 10;12(1):e0169609. doi: 10.1371/journal.pone.0169609 (PMC5224795; doi:10.1371/journal.pone.0169609)
Supplement: S1 File — Statistical analysis of dose- and time-dependent phosphorylation of Akt in platelets on SDF-1α treatment (Table A). Statistical analysis of effect of CXCR4 antagonist and PI3 kinase inhibitor on SDF-1α-induced Akt phosphorylation (Table B). Statistical analysis of inhibition of SDF-1α-induced Akt phosphorylation by raft disruption with methyl-β-cyclodextrin (Table C). (PDF) [file pone.0169609.s001.pdf]

Table A

Comparison	*P* (Thr308)	*P* (Ser473)
0 ng/ml versus 40 ng/ml	0.4274	0.9047
0 ng/ml versus 200 ng/ml	0.088	0.208
0 ng/ml versus 1000 ng/ml	0.0605	0.0516
0 ng/ml versus 4000 ng/ml	0.0115*	0.0011*
40 ng/ml versus 4000 ng/ml	0.1781	0.0034*
200 ng/ml versus 4000 ng/ml	0.6854	0.0315*
0 min versus 2 min	0.5828	0.0402*
0 min versus 5 min	0.2274	0.0069*
0 min versus 10 min	0.0132*	0.0002*
10 min versus 30 min	0.0212*	0.0135*

*Statistically significant at $P<0.05$

Table B

Comparison	*P* (Thr308)	*P* (Ser473)
Resting versus SDF-1α	0.0027*	0.0006*
SDF-1α versus AMD3100 + SDF-1α	0.0003*	0.0002*
SDF-1α versus LY294002 + SDF-1α	<.0001*	0.0002*
Resting versus AMD3100 + SDF-1α	0.2847	0.517
Resting versus LY294002 + SDF-1α	0.0081*	0.6754
AMD + SDF-1α versus LY + SDF-1α	0.1158	0.9912

*Statistically significant at $P<0.05$

Table C

Comparison	*P* (Thr308)	*P* (Ser473)
Resting versus SDF-1α	<.0001*	0.002*
SDF-1α versus MβCD + SDF-1α	<.0001*	0.0063*
Resting versus MβCD + SDF-1α	0.0244*	0.4825

*Statistically significant at $P<0.05$
